# Incidence and influencing factors of chemotherapy-induced peripheral neuropathy in cancer patients: a systematic review and meta-analysis

**DOI:** 10.3389/fneur.2026.1672180

**Published:** 2026-02-24

**Authors:** He Yan, Yan Dan, Li Nana, Guo Dongqing, Zhang Junming, Chu Xin

**Affiliations:** 1 School of Nursing, Chengdu University of Traditional Chinese Medicine, Chengdu, China; 2 Nursing Department, Hospital of Chengdu University of Traditional Chinese Medicine, Chengdu, China

**Keywords:** cancer patients, chemotherapy, influencing factors, meta-analysis, peripheral neuropathy

## Abstract

**Background:**

Chemotherapy-induced peripheral neuropathy (CIPN) is a common adverse effect in cancer patients, yet studies have reported inconsistent incidence and influencing factors for CIPN. This study aims to systematically evaluate the incidence and influencing factors of CIPN in cancer patients.

**Methods:**

The search databases included PubMed, Embase, Cochrane Library, and Web of Science, focusing on CIPN incidence and influencing factors in cancer patients up to 10 June, 2025. A meta-analysis of incidence and influencing factors was performed separately by Stata 17.0 software.

**Results:**

23 studies involving a sample size of 15,090 cases were included, and 16 influencing factors were identified. The incidence of CIPN in cancer patients was 56% (95% CI: 46–66%, *p* < 0.01). Age ≥50 years (*OR* = 1.07, 95% CI: 1.03–1.10), BMI ≥ 24 kg/m^2^ (*OR* = 1.15, 95% CI: 1.06–1.24), BMI ≥ 30 kg/m^2^ (*OR* = 1.67, 95% CI: 1.43–1.95), anxiety or depression (*OR* = 2.50, 95% CI: 1.20–5.20), hypertension (*OR* = 1.98, 95% CI: 1.07–3.69), diabetes mellitus (*OR* = 1.66, 95% CI: 1.29–2.13), cumulative dose of chemotherapy drugs (*OR* = 2.52, 95% CI: 1.20–5.32), chemotherapy≥4 cycles (*OR* = 1.21, 95% CI: 1.08–1.35), combination with taxane chemotherapy (per 10 mg/m^2^) (*OR* = 3.14, 95% CI: 1.81–5.44), vitamin D deficiency (*OR* = 5.63, 95%CI: 2.64–11.99), high cholesterol (*OR* = 1.34, 95% CI: 1.14–1.58), and transaminase elevation (*OR* = 2.10, 95% CI: 1.55–2.84) were influencing factors for CIPN in cancer patients.

**Conclusion:**

The results show that the incidence of CIPN in cancer patients is at a high level, and its influencing factors are varied and complex. This suggests that clinicians should screen for CIPN early to improve clinical outcomes and enhance the quality of life and survival for cancer patients.

**Systematic review registration:**

https://www.crd.york.ac.uk/PROSPERO/, CRD420251010306.

## 1 Introduction

The latest data from the WHO/International Agency for Research on Cancer (IARC) in Global Cancer Statistics 2022 indicates that malignant tumors represent a significant global public health challenge, resulting in nearly 10 million fatalities and approximately 20 million new cancer cases worldwide in 2022 (1). In 2022, the cancer incidence rate in China was 341.75 per 100,000, while the mortality rate was 182.34 per 100,000 (2). Furthermore, Cancer Statistics, 2025 predicts that by 2025, the cancer incidence and mortality rates in the United States will reach 620.5 per 100,000 and 187.6 per 100,000, respectively (3). Chemotherapy is a systemic anti-tumor treatment that has become one of the standard methods of anti-tumor therapy. It is applicable not just for consolidation therapy following surgery and during radiotherapy, but also particularly for malignant tumors that are inoperable or have metastasized (4). The global statistics analysis indicates that from 2018 to 2040, 15 million cancer patients will receive chemotherapy for the first time worldwide, and the global chemotherapy utilization rate will be approximately 57.7% (5). The rising number of chemotherapy patients exacerbates the unpleasant reactions linked to treatment, significantly affecting patients’ health and quality of life, with CIPN being one of the most common adverse effects (6).

CIPN is a common dose-limiting adverse reaction in cancer chemotherapy patients, resulting in sensory, motor, and autonomic nerve damage, manifesting as pain, numbness, balance disorders, limb weakness, and increased risk of falling, which affects patients’ quality of life (7, 8). In 2020, the American Society of Clinical Oncology (ASCO) updated its guidelines for the prevention and treatment of CIPN. They emphasize that when patients exhibit significant neuropathy or functional impairment, physicians must swiftly evaluate the necessity for treatment delay, dose modification, substitution, or cessation of chemotherapy, as this will critically impact the efficacy of anti-tumor therapy (9).

CIPN has become a pressing public health issue due to the rising number of chemotherapy patients and tumor incidence each year. Its incidence and influencing factors have also gained significant attention from academics both domestically and internationally. Currently, the majority of domestic and international research on the overall incidence of CIPN is characterized by single-center studies with limited sample sizes, revealing incidence rates that vary from 16.9 to 89.4%, exhibiting considerable discrepancies (10). In addition, due to the different research times and regions, there were inconsistent research conclusions on the influencing factors related to CIPN. As a result, this study will provide an evidence summary of the incidence and influencing factors of CIPN in cancer patients. This evidence summary seeks to elevate knowledge of CIPN among clinical healthcare professionals and establish a basis for screening the factors influencing CIPN in high-risk populations, with the objective of diminishing the incidence of CIPN in cancer patients.

## 2 Materials and methods

### 2.1 Protocol registration

This study followed the PRISMA guidelines for systematic review and meta-analysis (11) and was registered on PROSPERO (registration No: CRD420251010306).

### 2.2 Search strategies

The search databases included PubMed, Embase, Cochrane Library, and Web of Science, from inception to 10 June, 2025. Search terms included Neoplasms, Cancer, Malignant Neoplasm, Chemotherapy, Maintenance Chemotherapy, Induction Chemotherapy, Chemotherapy-Induced Peripheral Neuropathy, Peripheral Neuropathies, and Peripheral Nervous System Diseases. The complete search strategy is shown in Supplementary Tables 1–4.

### 2.3 Selection criteria

Inclusion criteria: (1) Patients with confirmed malignant tumors and chemotherapy; (2) The study design included cross-sectional studies, case–control studies, and cohort studies; (3) The results of the study included incidence rate of CIPN and related factors, where the influencing factors needed to be included in the relevant data provided by the study or could be converted to a 95% CI and odds ratio (OR).

Exclusion criteria: (1) reviews, conference papers, systematic reviews; (2) duplicate reported studies; (3) studies with unavailability of full text; (4) studies with low quality evaluation.

### 2.4 Study screening and data extraction

After removing duplicates from all the literature using EndNote 21 software, the two researchers separately reviewed the titles and abstracts, then engaging in full-text examination and data extraction. If there were any discrepancies in this process, the decision was made by a third researcher after assessment. The extracted information included: first author, year of publication, country, study type, sample size, and CIPN incidence and influencing factors.

### 2.5 Quality assessment

Two researchers assessed the quality of the studies, with differences resolved by a third researcher’s evaluation. The Agency for Healthcare Research and Quality tool (AHRQ) was used to evaluate the quality of the cross-sectional studies, with a total score of 11, 8–11, 4–7, and 0–3 corresponding to high, medium, and low quality (12). The Newcastle-Ottawa Scale (NOS) was used to evaluate the quality of cohort and case–control studies, with a total score of 9, 7–9, 5–6, and 0–4 corresponding to high, medium, and low quality literature (13).

### 2.6 Statistical analysis

Statistical analysis of the incidence and influencing factors of CIPN in cancer patients utilized Stata 17.0 software. A random-effects model was used for high heterogeneity (*I^2^* ≥ 50% and *p* ≤ 0.10); conversely, a fixed-effects model was used for moderate or low heterogeneity (*I^2^* < 50% and *p* > 0.10). Funnel plots were drawn for more 10 studies included in the literature, and the Egger’s test was used to test for publication bias, and if publication bias existed, the results were judged by trim-and-fill tests.

## 3 Results

### 3.1 Study screening results

**Figure 1:**
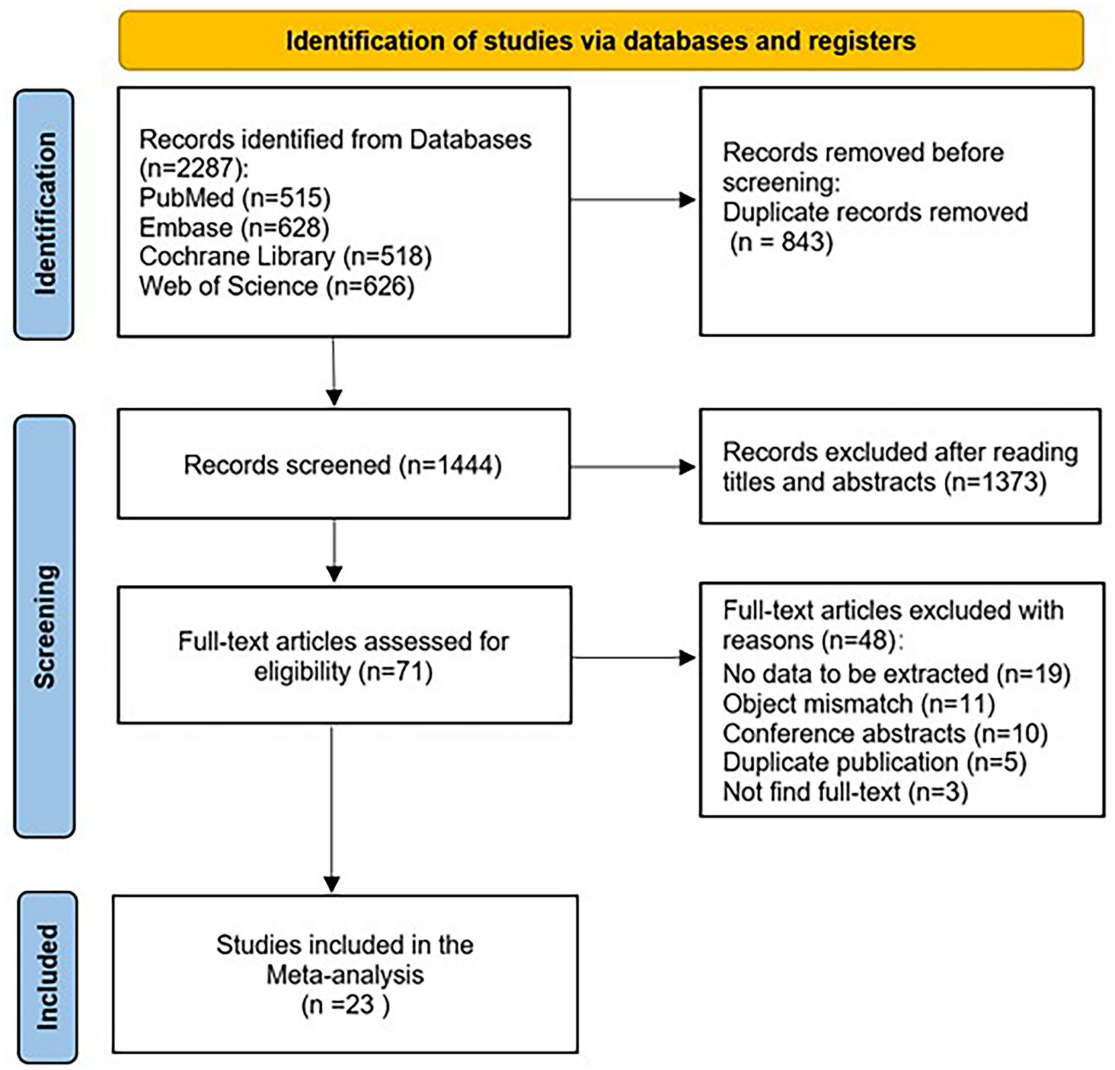
PRISMA flowchart.

From a total of 2,287 studies retrieved from the database, 843 duplicates were excluded, 1,373 were excluded after reviewing titles and abstracts, 71 were read in full text, and ultimately, 23 were included. The results of the literature screening are shown in Figure 1.

### 3.2 Study characteristics

There are 23 studies with a sample size of 15,090 cases from 10 different countries that were included. The incidence of CIPN in these studies ranged from 17 to 84%. Influential factors for CIPN inclusion were required to be present in ≥2 studies, and 16 influential factors were extracted. The characteristics included in the study are shown in Table 1.

**Table 1:** Characteristics of included studies.

Study	Year	Country	Study type	Case/sample size	Incidence (%)	Influencing factors	Quality assessment
Sun et al. (40)	2024	China	Cohort study	277/350	0.79	a, c, e, f, h, j, n	7^2^
Liang et al. (42)	2024	China	Cohort study	195/268	0.73	c, h, n	7^2^
Kuguyo et al. (43)	2024	Zimbabwe	Cohort study	176/252	0.70	k, m	7^2^
Hiramoto et al. (44)	2024	Japan	Cohort study	238/283	0.84	a, n	8^2^
Habtie et al. (45)	2024	Ethiopia	Cross-sectional studies	211/406	0.54	f, k, o, p	8^1^
Zhi et al. (46)	2023	American	Cohort study	–	–	c	6^2^
Sreeram et al. (47)	2023	American	Cohort study	704/1034	0.68	l	7^2^
Dorand et al. (48)	2023	American	Cohort study	1104/3387	0.33	b, c, d, g, n, o	8^2^
Timmins et al. (49)	2022	Australia	Cross-sectional studies	296/379	0.78	a	9^1^
Kanbayashi et al. (50)	2022	Japan	Cohort study	60/76	0.79	a, c	6^2^
Catalano et al. (51)	2022	Italy	Cohort study	47/153	0.31	a, o	6^2^
Trendowski et al. (14)	2021	American	Cross-sectional studies	550/1045	0.53	c, e, i	8^1^
Mizrahi et al. (52)	2021	Australia	Cohort study	238/333	0.72	a, b, c	6^2^
Kamgar et al. (53)	2021	American	Cohort study	401/2420	0.17	c, p	7^2^
Huang et al. (54)	2021	China	Case–control study	–	–	g	6^2^
Tsurutani et al. (55)	2019	Japan	Cohort study	105/374	0.28	n	7^2^
Molassiotis et al. (56)	2019	Singapore, Britain	Cohort study	182/255	0.71	a, o	6^2^
Kolb et al. (57)	2019	American	Cohort study	413/565	0.73	f, g, i, j	6^2^
Lee et al. (58)	2018	Korea	Cohort study	50/111	0.45	e	5^2^
Bandos et al. (59)	2018	American	Cohort study	635/1512	0.42	a, c, d, l	7^2^
Hershman et al. (25)	2016	American	Cohort study	372/1401	0.27	a, g, p	6^2^
Bao et al. (60)	2016	American	Cross-sectional studies	173/296	0.58	d	7^1^
Kanbayashi et al. (61)	2010	Japan	Cohort study	75/190	0.39	m, o	6^2^

a Age ≥50 years, b Female, c BMI≥24 kg/m^2^, d BMI≥30 kg/m^2^, e Anxiety or depression, f Hypertension, g Diabetes mellitus, h Vitamin D deficiency, i High cholesterol, j Transaminase elevation, k Advanced stage of the tumor, l Breast cancer at primary site, m Combined pain medications, n Cumulative dose of chemotherapy drugs, o Chemotherapy ≥4 cycles, p Combination with taxane chemotherapy (per 10 mg/m^2^); − indicates no relevant data in the study. ^1^The AHRQ score. ^2^The NOS score.

### 3.3 Quality assessment

The methodological quality assessment of 23 studies is shown in Table 1. In the cross-sectional study, two studies had a quality score of 8, and the remaining two studies had quality scores of 7 and 9, respectively. In the cohort study and case–control study, the two studies’ quality scores were 8, seven studies’ quality scores were 7, nine studies’ quality scores were 6, and one study’s quality score was 5.

### 3.4 Results

#### 3.4.1 Incidence of CIPN in cancer patients

**Figure 2:**
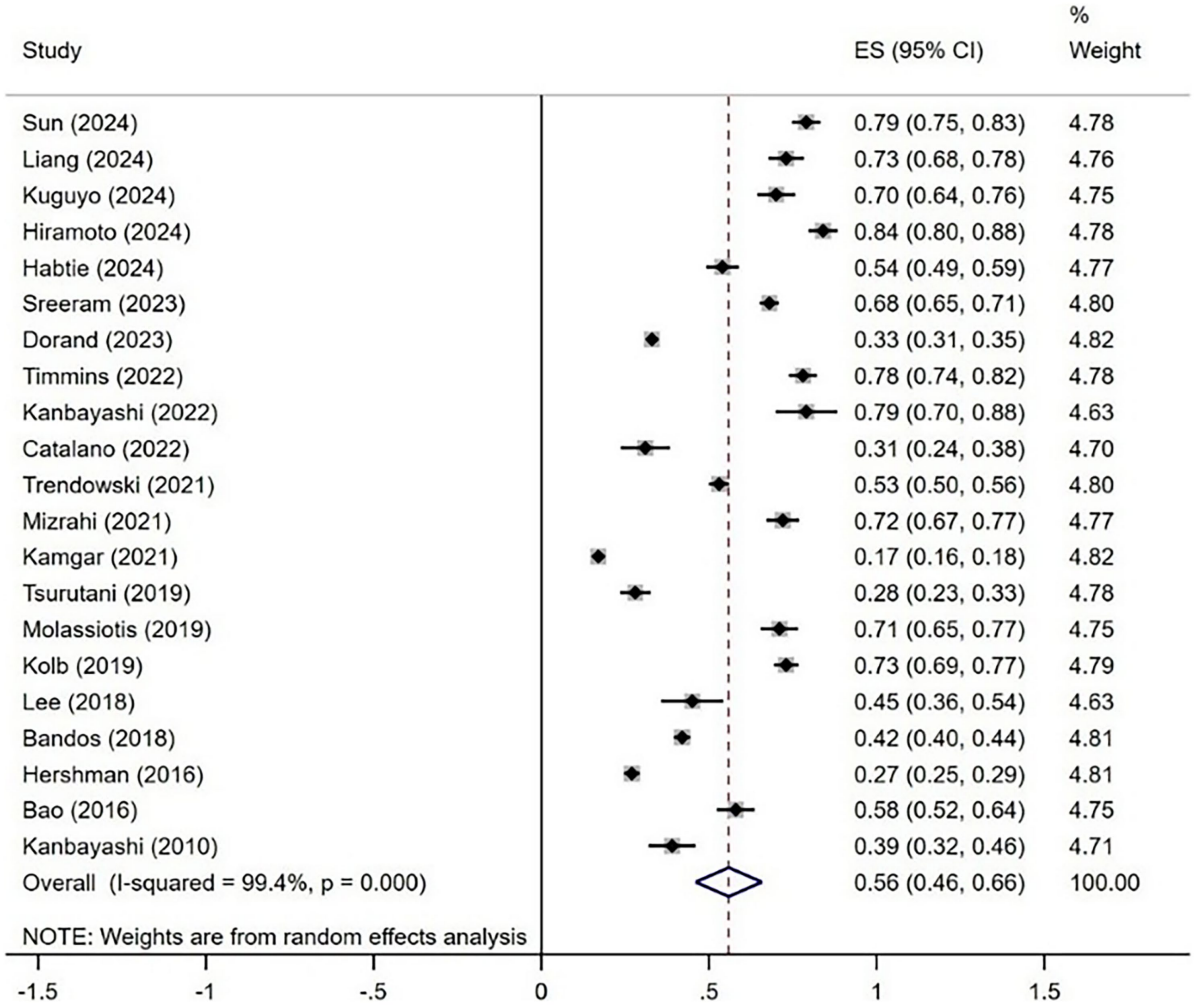
Forest plot of CIPN incidence.

The meta-analysis included 21 studies that provided the incidence of CIPN. The merged effect sizes showed that the incidence of CIPN among cancer patients was 56% (95% CI: 46% ~ 66%, *I*^2^ = 99.4%, *p* < 0.01), and the random effects model was chosen. The forest plot is shown in Figure 2.

#### 3.4.2 Influential factors related to CIPN in cancer patients

In 23 studies, the influencing factors of CIPN had to be present in ≥ 2 studies, and a total of 16 relevant influencing factors were extracted. The results of Meta-analysis showed that individual factors (age ≥50 years, BMI ≥ 24 kg/m^2^, BMI ≥ 30 kg/m^2^), disease-related factors (anxiety or depression, hypertension, diabetes mellitus), drug-related factors [cumulative dose of chemotherapy drugs, chemotherapy ≥4 cycles, combination with taxane chemotherapy (per 10 mg/m^2^)], and physiological indicators (vitamin D deficiency, high cholesterol, and transaminase elevation) are the influencing factors of CIPN in cancer patients. There was insufficient evidence of an association between the other four influencing factors (female, advanced stage of the tumor, breast cancer at primary site, and combined pain medications) and CIPN. The specific results are shown in Table 2.

**Table 2:** Meta-analysis of factors influencing CIPN.

Influence factors	Number of studies	Heterogeneity test results		Effect model	Meta-analysis results	
		*I^2^*(%)	*P*		OR (95% CI)	*p*-value
Individual factors						
Age≥50 years	9	68	0.002	Random	1.07(1.03, 1.10)	0.000
Female	2	90	0.002	Random	0.70(0.19, 2.55)	0.590
BMI ≥ 24 kg/m^2^	9	81	0.000	Random	1.15(1.06, 1.24)	0.000
BMI ≥ 30 kg/m^2^	3	24	0.269	Fixed	1.67(1.43, 1.95)	0.000
Disease-related factors						
Anxiety or depression	3	52	0.100	Random	2.50(1.20, 5.20)	0.014
Hypertension	3	64	0.062	Random	1.98(1.07, 3.69)	0.030
Diabetes mellitus	4	52	0.100	Random	1.66(1.29, 2.13)	0.000
Advanced stage of the tumor	2	85	0.010	Random	2.19(0.68, 7.07)	0.192
Breast cancer at primary site	2	88	0.003	Random	2.21(0.81, 6.02)	0.120
Drug-related factors						
Combined pain medications	2	76	0.040	Random	1.99(0.70, 5.69)	0.199
Cumulative dose of chemotherapy drugs	5	88	0.000	Random	2.52(1.20, 5.32)	0.015
Chemotherapy ≥4 cycles	5	73	0.006	Random	1.21(1.08, 1.35)	0.001
Combination with taxane chemotherapy (per 10 mg/m^2^)	3	71	0.031	Random	3.14(1.81, 5.44)	0.000
Physiological indicators						
Vitamin D deficiency	2	0	0.761	Fixed	5.63(2.64, 11.99)	0.000
High cholesterol	2	0	0.901	Fixed	1.34(1.14, 1.58)	0.001
Transaminase elevation	2	0	0.468	Fixed	2.10(1.55, 2.84)	0.000

### 3.5 Sensitivity analysis

**Figure 3:**
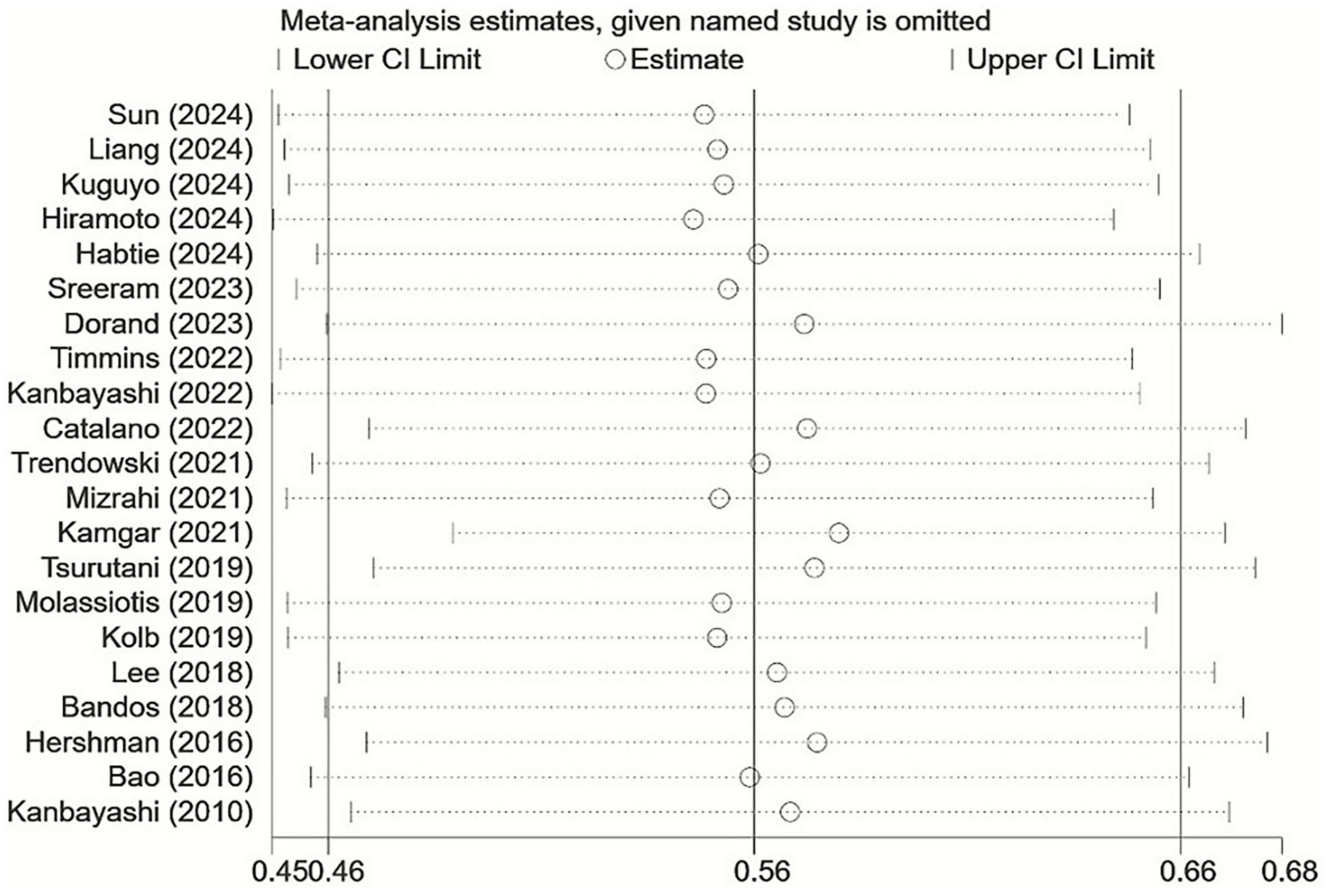
Sensitivity analysis of CIPN incidence.

The one-by-one elimination method was used to conduct a sensitivity analysis on the incidence of CIPN in cancer patients, revealing no significant change in the combined effect sizes. This indicates that meta-analysis results were relatively stable, as illustrated in Figure 3. The sensitivity analysis of the influencing factors of CIPN in cancer patients was performed by converting different effect models, the results showed that the OR, 95% CI and *p*-values of most CIPN influencing factors were similar before and after the conversion of effect models, as shown in Supplementary Table 5.

### 3.6 Publication bias

**Figure 4:**
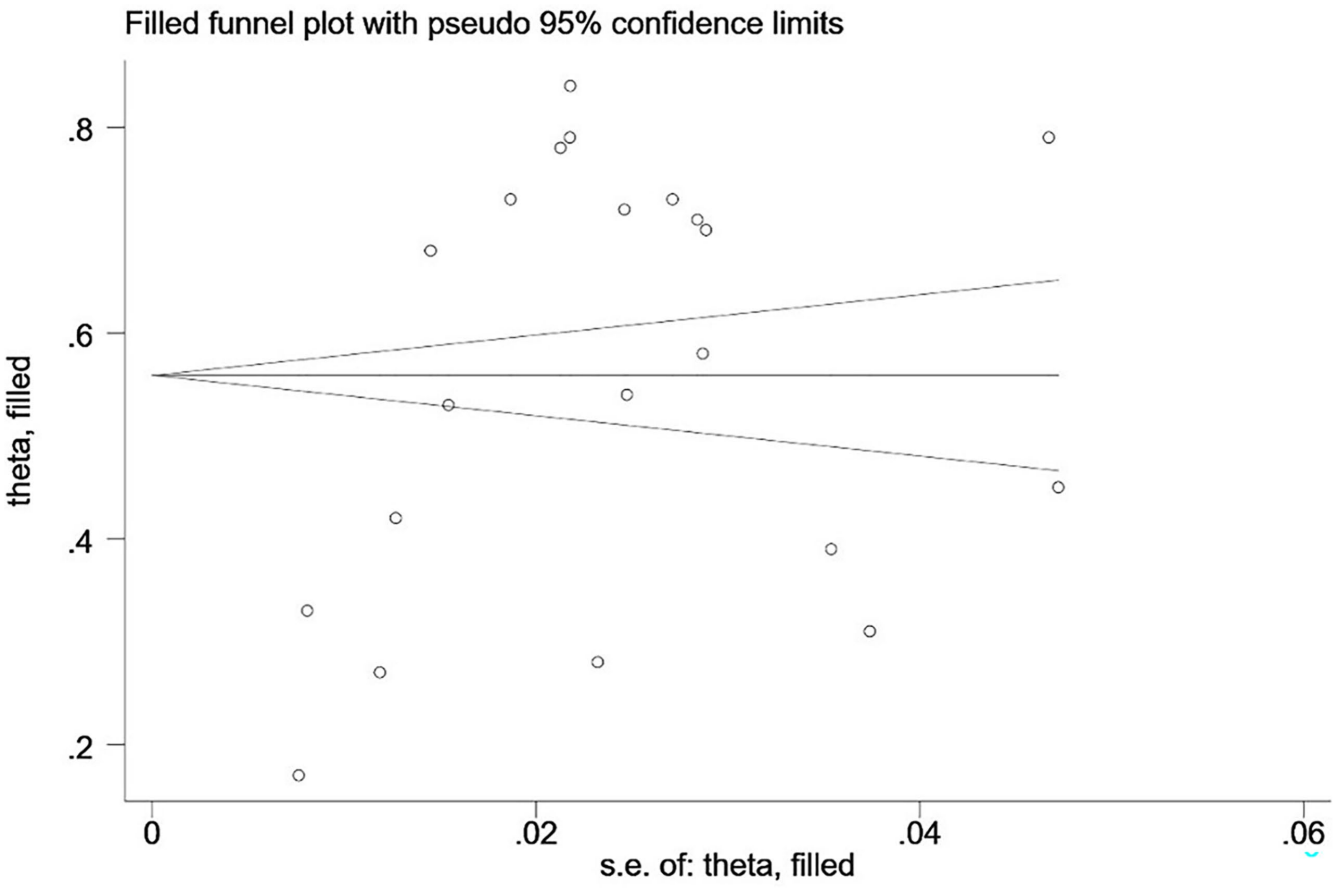
Trim-and-fill tests.

The incidence of CIPN in cancer patients was plotted on a funnel plot, revealing asymmetry. Egger’s test suggests *p* < 0.05, indicating publication bias in the study. Validation through the trim-and-fill tests showed that no studies were added, indicating that publication bias had no significant impact on the study results. The conclusions remain reliable, as shown in Figure 4.

## 4 Discussion

### 4.1 Incidence of CIPN in cancer patients

CIPN is among the most common adverse reactions of chemotherapy in cancer patients, significantly affecting their daily lives, psychological well-being, and treatment efficacy (14, 15). However, the reported prevalence of CIPN varied across studies due to changes in demographic, sample size, and study design. This meta-analysis included 21 studies from 10 countries, revealing the incidence of CIPN among cancer patients of 56% (95% CI: 46–66%), which was higher than reported in the majority of the included studies. Furthermore, the global cancer burden will persistently increase due to population aging, chronic infections, smoking, air pollution, and other tumor risk factors, indicating a necessity for additional clinical intervention studies on CIPN in cancer patients to alleviate the disease burden (16). The sensitivity analysis of the study showed that the results were relatively stable.

In recent years, drug therapy has remained the main approach to managing symptoms related to CIPN. Currently, systemic treatments widely used for CIPN include anticonvulsants, antidepressants, and opioids. Studies have shown that anticonvulsants such as gabapentin and pregabalin can reduce Ca^2+^ influx, effectively controlling the pathological pain of CIPN (17). According to the clinical practice guidelines of the American Society of Clinical Oncology, duloxetine is recommended for treating CIPN (18). Research indicates that duloxetine alleviates pain by inhibiting the MAPK signaling pathway, preventing the translocation of NF-κB into the nucleus, and reducing the production of inflammatory mediators such as tumor necrosis factor, thereby easing the inflammatory response (19). Opioids have significant analgesic effects, but due to the risks of misuse and addiction, they are not recommended as a first-line treatment (20). In addition, non-pharmacological management strategies are increasingly being recognized for alleviating symptoms related to CIPN. Studies have shown that cryotherapy in physical therapy can limit the release of neurotoxic drugs to peripheral nerves, reducing the incidence and severity of CIPN (21). Acupuncture interventions can promote adrenaline transmission, activate the serotonin system, reduce nerve sensitivity, and improve patients’ quality of life (22). Exercise interventions enhance blood metabolic circulation in tissues, accelerate the repair of damaged nerves, promote the growth of nerve factors, and improve nerve function (23). However, their advantages are more evident in relieving cancer-related fatigue, regulating psychosocial function, and improving sleep quality (24). Therefore, future research needs to further explore interventions that can significantly improve clinical symptoms in CIPN patients and enhance treatment adherence, thereby reducing disease burden and improving quality of life.

### 4.2 Influencing factors of CIPN in cancer patients

#### 4.2.1 Individual factors

The results revealed that age ≥50 years, BMI ≥ 24 kg/m^2^, and BMI ≥ 30 kg/m^2^ were influencing factors of CIPN in cancer patients. Studies have shown that the incidence of CIPN in older adults treated with paclitaxel increases by 4% for each year of age (25). The diminished metabolic rate, neurological nutritional deficits, and reduced cellular repair capability in older individuals may heighten their vulnerability to CIPN as they age (26). In addition, cancer patients with a BMI of ≥24 kg/m^2^and ≥ 30 kg/m^2^will have an increased risk of CIPN, and those with a BMI ≥ 30 kg/m^2^face an even higher risk, potentially due to the higher adiposity in overweight or obese individuals compared to those with normal body weight. Adiposity plays an important role in mediating neurological damage. Simultaneously, the majority of chemotherapy agents are classified as lipophilic pharmaceuticals; thus, individuals with elevated body fat may experience prolonged exposure to neurotoxic medications, resulting in an increased incidence of CIPN (27). It recommends that clinicians personalize chemotherapy drug use to lower the incidence of CIPN.

#### 4.2.2 Disease-related factors

The results showed that anxiety or depression, hypertension, and diabetes mellitus were influencing factors for CIPN in cancer patients. Studies demonstrate that individuals with anxiety or depression may elevate inflammatory cytokines, potentially impairing the repair mechanisms following nerve injury (28). And the lower immunity and reduced 5-hydroxytryptamine in the brain of this group of patients will be more sensitive to chemotherapy stimuli, so the incidence of CIPN is higher (29). Hypertension can disrupt autonomic control, and sustained elevated blood pressure may result in enduring damage to peripheral blood vessels, creating a detrimental cycle that exacerbates the incidence of CIPN (30). Due to the disorders of glucose metabolism in diabetic patients, its complications can spread throughout the important organs and blood vessels of the whole body, which in itself can lead to damage of peripheral nerves, and the use of chemotherapy drugs will further damage nerve function, which aggravates the occurrence of CIPN (31). In addition, research indicates that diabetic patients are two-thirds as likely to experience CIPN compared to non-diabetic patients (25). Consequently, individuals with anxiety or depression ought to receive enhanced familial and partner support to mitigate their adverse emotions (32), actively regulate blood pressure and blood glucose levels within the normative range, and develop tailored dietary and exercise regimens to minimize impairment to peripheral nerve function.

#### 4.2.3 Drug-related factors

Our study showed that the cumulative dose of chemotherapy drugs, chemotherapy ≥4 cycles, and combination with taxane chemotherapy (per 10 mg/m^2^) were the influencing factors of CIPN in cancer patients. The study showed that the incidence of CIPN in patients undergoing more than four rounds of chemotherapy was markedly greater than in those receiving fewer than four cycles, corroborating the findings of this research (33). Since CIPN is a dose-limiting toxicity of chemotherapy, the greater the cumulative dose of drug infusion, the greater the damage to peripheral nerves, and the incidence of CIPN increases (10, 34). Our study also indicated that the combination with taxane chemotherapy (per 10 mg/m^2^) is a significant factor for CIPN. Paclitaxel-based chemotherapy is a highly recommended clinical first-line drug worldwide, but the mechanism between taxane chemotherapy and CIPN is still unclear (35). Some studies have also suggested that taxanes may impair mitochondrial DNA function and increase oxidative stress. In addition, taxanes can directly damage myelin sheaths to impair neurotransmission, which further contributes to the development of CIPN (36, 37). These indicate that the clinic must rigorously regulate the dosage and treatment cycles while administering neurotoxic agents to reduce the incidence of CIPN.

#### 4.2.4 Physiological indicators

Meta-analysis showed that vitamin D deficiency, high cholesterol, and elevated aminotransferases were the influencing factors of CIPN in cancer patients. Research indicates that patients with vitamin D insufficiency prior to chemotherapy have a higher prevalence of CIPN, more severe symptoms, and a three-fold increase in the likelihood of treatment cessation (38). It may be that vitamin D, by regulating the level of epidermal factors, can promote the growth and differentiation of nerve cells and have a protective and reparative effect on the peripheral nervous system, so the incidence of CIPN is higher in patients with vitamin D deficiency (39). Currently, there are no studies to confirm the direct relationship between high cholesterol and CIPN. It is speculated that high cholesterol can cause a series of complications such as obesity, hypertension, and diabetes, which in turn are closely related to the occurrence of CIPN. Furthermore, the majority of chemotherapeutic agents metabolized by the liver inflict direct harm to hepatocytes, leading to hepatic dysfunction and elevated transaminase levels. Concurrently, compromised hepatocytes extend the elimination duration of these drugs, and the prolonged retention of chemotherapeutic agents in the organism exacerbates peripheral nervous system damage, thereby heightening the incidence of CIPN (40, 41).

## 5 Limitations

Our study has limitations as follows: (1) although sensitivity analysis indicates study results are stable, there is still a certain degree of publication bias, probably because only Chinese or English studies was included, and there is a certain degree of language bias or publication bias; (2) the results of individual influencing factors were unstable, and the credibility needs to be verified; (3) some influencing factors were included in a small number of studies, which may affect meta-analysis results; (4) due to the different influencing factors, some influencing factors cannot be combined, which may lead to the incomplete analysis of the research results. Ultimately, owing to constraints in study quality and quantity, our conclusions necessitate validation through studies with larger samples and higher quality.

## 6 Conclusion

In summary, the incidence of CIPN remains relatively high, and the following risk factors contribute to it: age ≥50 years, BMI ≥ 24 kg/m^2^, BMI ≥ 30 kg/m^2^, anxiety or depression, hypertension, diabetes mellitus, cumulative dose of chemotherapy drugs, chemotherapy≥4 cycles, combination with taxane chemotherapy (per 10 mg/m^2^), vitamin D deficiency, high cholesterol, and elevated transaminases. Clinical medical personnel must recognize the risk factors for CIPN early and act swiftly, as this is vital for cancer patients to improve the efficacy of chemotherapy and their quality of survival.

## Data Availability

The original contributions presented in the study are included in the article/Supplementary material, further inquiries can be directed to the corresponding author.
