# Supplementary material for: Incidence and influencing factors of chemotherapy-induced peripheral neuropathy in cancer patients: a systematic review and meta-analysis
Source: Front Neurol. 2026 Feb 24;17:1672180. doi: 10.3389/fneur.2026.1672180 (PMC12971429; doi:10.3389/fneur.2026.1672180)
Supplement: Supplementary file 1 [file Table_1.docx]

**Table S1 ： PubMed search strategy**

| Steps | Literature search queries |
| --- | --- |
| #1 | ("Neoplasms"[Mesh]) OR (Cancer[Title/Abstract] OR Cancers[Title/Abstract] OR Malignant Neoplasm[Title/Abstract] OR Malignancies[Title/Abstract] OR Malignant Neoplasms[Title/Abstract] OR Neoplasms, Malignant[Title/Abstract]) |
| #2 | ((("Consolidation Chemotherapy"[Mesh]) OR "Induction Chemotherapy"[Mesh]) OR "Maintenance Chemotherapy"[Mesh]) OR (Chemotherapy[Title/Abstract] OR chemotherapeutic[Title/Abstract] OR During chemotherapy[Title/Abstract]) |
| #3 | ("Peripheral Nervous System Diseases"[Mesh]) OR (Peripheral Nervous System Disorders[Title/Abstract] OR Peripheral Neuropathy[Title/Abstract] OR Peripheral Nervous System Disease[Title/Abstract] OR Chemotherapy-Induced Peripheral Neuropathy[Title/Abstract] OR chemotherapy induced peripheral neuropathy[Title/Abstract] OR CIPN[Title/Abstract]) |
| #4 | ((((Influencing factors[Title/Abstract]) OR (Risk factors[Title/Abstract])) OR (Predictors[Title/Abstract])) OR (Related factors[Title/Abstract])) OR (factors[Title/Abstract]) |
| #5 | #1 AND #2 AND #3 AND #4 |

**Table S2 ： Embase search strategy**

| Steps | Literature search queries |
| --- | --- |
| #1 | 'malignant neoplasm'/exp |
| #2 | 'cancer':ti,ab,kw OR 'cancers':ti,ab,kw OR 'malignant neoplasia':ti,ab,kw OR 'malignant neoplastic disease':ti,ab,kw OR 'malignant tumor':ti,ab,kw OR 'malignant tumour':ti,ab,kw OR 'neoplasia, malignant':ti,ab,kw OR 'neoplasmic malignancy':ti,ab,kw OR 'neoplastic malignancy':ti,ab,kw OR 'oncologic malignancy':ti,ab,kw OR 'oncological malignancy':ti,ab,kw OR 'tumor, malignant':ti,ab,kw OR 'tumoral malignancy':ti,ab,kw OR 'tumorous malignancy':ti,ab,kw OR 'tumour, malignant':ti,ab,kw OR 'malignant neoplasm':ti,ab,kw |
| #3 | #1 OR #2 |
| #4 | 'chemotherapy'/exp |
| #5 | 'chemotherapeutic medication':ti,ab,kw OR 'chemotherapeutic treatment':ti,ab,kw OR 'chemotherapeutics':ti,ab,kw OR 'chemotherapy':ti,ab,kw |
| #6 | #4 OR #5 |
| #7 | 'peripheral neuropathy'/exp |
| #8 | 'neuropathy, peripheral':ti,ab,kw OR 'peripheral nerve disease':ti,ab,kw OR 'peripheral nerve disorder':ti,ab,kw OR 'peripheral nervous disease':ti,ab,kw OR 'peripheral nervous system disease':ti,ab,kw OR 'peripheral nervous system diseases':ti,ab,kw OR 'peripheral nervous system disorder':ti,ab,kw OR 'pns disease':ti,ab,kw OR 'pns disorder':ti,ab,kw OR 'peripheral neuropathy':ti,ab,kw |
| #9 | #7 OR #8 |
| #10 | 'influencing factors':ti,ab,kw OR 'related factors':ti,ab,kw OR 'risk factors':ti,ab,kw OR 'predictors':ti,ab,kw |
| #11 | #3 AND #6 AND #9 AND #10 |

**Table S3 ：Cochrane library search strategy**

| Steps | Literature search queries |
| --- | --- |
| #1 | MeSH descriptor: [Neoplasms] explode all trees |
| #2 | (Neoplasias OR Neoplasia OR Tumors OR Tumor OR Neoplasm OR Malignancies OR Neoplasms, Malignant OR Malignancy OR Cancer OR Malignant Neoplasm OR Cancers OR Neoplasm, Malignant OR Malignant Neoplasms OR Neoplasms, Benign OR Benign Neoplasm OR Benign Neoplasms OR Neoplasm, Benign):ti,ab,kw |
| #3 | #1 OR #2 |
| #4 | MeSH descriptor: [Drug Therapy] explode all trees |
| #5 | (Therapy, Drug OR Pharmacotherapies OR Pharmacotherapy OR Chemotherapy OR Chemotherapies OR Therapies, Drug OR Drug Therapies):ti,ab,kw |
| #6 | #4 OR #5 |
| #7 | MeSH descriptor: [Peripheral Nervous System Diseases] explode all trees |
| #8 | (Peripheral Nerve Diseases OR Peripheral Nerve Disease OR Peripheral Nervous System Disorders OR Nerve Diseases, Peripheral OR Nerve Disease, Peripheral OR PNS Diseases OR PNS Disease OR PNS (Peripheral Nervous System) Diseases OR Peripheral Nervous System Disease OR Neuropathy, Peripheral OR Peripheral Neuropathies OR Peripheral Neuropathy OR chemotherapy induced peripheral neuropathy OR Chemotherapy-Induced Peripheral Neuropathy OR CIPN):ti,ab,kw |
| #9 | #7 OR #8 |
| #10 | (Influencing factors OR Risk factors OR Predictors OR Related factors):ti,ab,kw |
| #11 | #3 AND #6 AND #9 AND #10 |

**Table S4 ：Web of science search strategy**

| Steps | Literature search queries |
| --- | --- |
| #1 | TS=(Neoplasms OR Cancer OR Cancers OR Malignant Neoplasm OR Malignancies OR Malignant Neoplasms OR Neoplasms, Malignant) |
| #2 | TS=(Consolidation Chemotherapy OR Induction Chemotherapy OR Maintenance Chemotherapy OR Chemotherapy OR chemotherapeutic OR During chemotherapy) |
| #3 | TS=(Peripheral Nervous System Diseases OR Peripheral Nervous System Disorders OR Peripheral Neuropathy OR Peripheral Nervous System Disease OR Chemotherapy-Induced Peripheral Neuropathy OR chemotherapy induced peripheral neuropathy OR CIPN) |
| #4 | AB=(Influencing factors OR Risk factors OR Predictors OR Related factors OR factors ) |
| #5 | #1 AND #2 AND #3 AND #4 |

**Table S5：Sensitivity analysis of influencing factors of CIPN**

| Influence factors | Random-effects model | |  | Fixed-effect model | | Stability |
| --- | --- | --- | --- | --- | --- | --- |
|  | *OR*(95%*CI*) | *P* |  | *OR*(95%*CI*) | *P* |  |
| Individual factors | | | | | | |
| Age≥50 years | 1.07(1.03,1.10) | < 0.001 |  | 1.06(1.05,1.08) | < 0.001 | stable |
| Female | 0.70(0.19,2.55) | 0.590 |  | 1.15(0.92,1.45) | 0.221 | stable |
| BMI≥24kg/m^2^ | 1.15(1.06,1.24) | < 0.001 |  | 1.03(1.02,1.05) | < 0.001 | stable |
| BMI≥30kg/m^2^ | 1.69(1.40,2.04) | < 0.001 |  | 1.67(1.43,1.95) | < 0.001 | stable |
| Disease-related factors | | | | | | |
| Anxiety or depression | 2.50(1.20,5.20) | 0.014 |  | 1.82(1.35,2.46) | < 0.001 | stable |
| Hypertension | 1.98(1.07,3.69) | 0.030 |  | 1.36(1.35,1.37) | < 0.001 | stable |
| Diabetes mellitus | 1.66(1.29,2.13) | < 0.001 |  | 1.57(1.35,1.81) | < 0.001 | stable |
| Advanced stage of the tumor | 2.19(0.68,7.07) | 0.192 |  | 1.35(1.16,1.57) | < 0.001 | instability |
| Breast cancer at primary site | 2.21(0.81,6.02) | 0.120 |  | 1.53(1.25,1.86) | < 0.001 | instability |
| Drug-related factors | | | | | | |
| Combined pain medications | 1.99(0.70,5.69) | 0.199 |  | 1.33(1.14,1.55) | < 0.001 | instability |
| Cumulative dose of chemotherapy drugs | 2.52(1.20,5.32) | 0.015 |  | 1.05(1.03,1.08) | < 0.001 | stable |
| Chemotherapy ≥ 4 cycles | 1.21(1.08,1.35) | 0.001 |  | 1.13(1.11,1.15) | < 0.001 | stable |
| Combination with taxane chemotherapy(per 10 mg/m^2^) | 3.14(1.81,5.44) | < 0.001 |  | 3.03(2.32,3.96) | < 0.001 | stable |
| Physiological indicators | | | | | | |
| Vitamin D deficiency | 5.63(2.64,11.99) | < 0.001 |  | 5.63(2.64,11.99) | < 0.001 | stable |
| High cholesterol | 1.34(1.14,1.58) | 0.001 |  | 1.34(1.14,1.58) | 0.001 | stable |
| Transaminase elevation | 2.10(1.55,2.84) | < 0.001 |  | 2.10(1.55,2.84) | < 0.001 | stable |
